# Pitch Discrimination Testing in Patients with a Voice Disorder

**DOI:** 10.3390/jcm11030584

**Published:** 2022-01-24

**Authors:** Duy Duong Nguyen, Antonia M. Chacon, Daniel Novakovic, Nicola J. Hodges, Paul N. Carding, Catherine Madill

**Affiliations:** 1 Voice Research Laboratory, Discipline of Speech Pathology, Faculty of Medicine and Health, The University of Sydney, Sydney, NSW 2006, Australia; antonia.chacon@sydney.edu.au (A.M.C.); daniel.novakovic@sydney.edu.au (D.N.); cate.madill@sydney.edu.au (C.M.); 2 National Hospital of Otorhinolaryngology, Hanoi 11519, Vietnam; 3 The Canterbury Hospital, Campsie, NSW 2194, Australia; 4 School of Kinesiology, University of British Columbia, Vancouver, BC V6T 1Z1, Canada; nicola.hodges@ubc.ca; 5 Faculty of Health and Life Sciences, Oxford Institute of Nursing, Midwifery and Allied Health Research, Oxford OX3 0BP, UK; pcarding@brookes.ac.uk

**Keywords:** auditory discrimination, voice control, voice assessment, voice disorders

## Abstract

Auditory perception plays an important role in voice control. Pitch discrimination (PD) is a key index of auditory perception and is influenced by a variety of factors. Little is known about the potential effects of voice disorders on PD and whether PD testing can differentiate people with and without a voice disorder. We thus evaluated PD in a voice-disordered group (*n* = 71) and a non-voice-disordered control group (*n* = 80). The voice disorders included muscle tension dysphonia and neurological voice disorders and all participants underwent PD testing as part of a comprehensive voice assessment. Percentage of accurate responses and PD threshold were compared across groups. The PD percentage accuracy was significantly lower in the voice-disordered group than the control group, irrespective of musical background. Participants with voice disorders also required a larger PD threshold to correctly discriminate pitch differences. The mean PD threshold significantly discriminated the voice-disordered groups from the control group. These results have implications for the voice control and pathogenesis of voice disorders. They support the inclusion of PD testing during comprehensive voice assessment and throughout the treatment process for patients with voice disorders.

## 1. Introduction

Laryngeal muscle control in voice production is affected by auditory feedback and sensorimotor reflexes [1]. There are overlapping anatomical pathways in the brain that encode similar acoustic information presented in both music and voice, such as waveform periodicity and amplitude envelope [2]. Coordination of laryngeal muscles in phonation depends upon motor planning, muscle activation, and feedback provided by auditory systems [1,3]. It has been demonstrated that disturbances in auditory perception/discrimination are related to problems within auditory motor reflexes governing effective laryngeal control. These perception problems lead to abnormal motor control patterns as observed in people with hyperfunctional dysphonia [4,5]. The impairment of temporal auditory function in patients with behavioral dysphonia may affect the success of voice therapy, suggesting the need for auditory processing assessment [6].

A disordered voice is defined as a voice that does not meet the occupational or social needs of the speaker and is inappropriate given the speaker’s age, gender, or situation [7]. Voice disorders can be classified according to the aetiology of the voice dysfunction [8]. Functional voice disorders include muscle tension voice disorder (MTVD) and psychogenic voice disorders [9]. Functional voice disorders may result from poor detection of pitch, volume, and voice quality dimensions in the absence of any neurological motor and sensory deficit [8,10]. In contrast, in neurological voice disorders, there is damage to the motor and/or sensory pathways. Distinguishing between different types of voice disorders requires not only voice quality assessment but also perception assessment, which allows conclusions to be made about the dependence of patient’s perception and vocal production upon specific sensory and motor pathways.

Auditory perception function can be evaluated using pitch discrimination (PD) testing. Pitch is a perceptual attribute of sound that has important roles in the human voice and PD is the ability to correctly detect intervals/differences between pitches of pure or complex tones. This ability to perceive different pitches is reflected in the experience of both perceiving and producing sound. PD also reflects auditory discrimination function. Tonal language speakers show greater pitch perception accuracy than non-tonal language speakers [11] and people with a musical background discriminate pitches more accurately than those with a non-musical background [12,13].

The neural processing of pitch is complicated. It involves hierarchical responses and mainly occurs in the right hemisphere of the brain; including the superior temporal gyrus, lateral Heschl’s gyrus, inferior frontal gyrus, insular cortex, and the inferior colliculus [14,15]. People possess variable pitch perception ability with some apparently having more difficulties in PD than others, probably due to their use of sub-optimal brain regions (e.g., left hemisphere) for pitch processing [16]. It was also found that there is differential neural pitch processing in the left and right hemispheres that allows the auditory system to detect temporal and spectral changes in the auditory feedback necessary for voice control [17]. PD is impaired in some congenital and acquired neurological conditions that involve organic neurological dysfunction. Congenital amusia (prevalence of 1.5%) [18] results in impaired pitch processing due to abnormal deactivation of the right inferior frontal gyrus [19]. Given that the auditory cortex shows normal responses to pitch in this condition, the suggestion has been made that the impairments are due to reduced white matter functional connections between the auditory and inferior frontal cortices [19]. Traumatic brain injury is also known to affect pitch perception ability due to damages of the underlying pitch processing regions [20].

Auditory discrimination problems have been shown in people with functional voice disorders. Abur et al. [4] showed that patients with hyperfunctional voice disorders had poorer auditory discrimination and more atypical adaptive responses to fundamental frequency (F0) shifts than those without the condition. Stepp et al. [5] showed that patients with hyperfunctional voice disorders demonstrated different patterns of adaptive responses in pitch perturbation tasks compared with controls. They suggested a disruption between auditory processing and laryngeal motor control. The pitch-shift reflex shows how well individuals can adapt their own pitch according to auditory feedback and has been examined in patients with muscle tension dysphonia (MTD) [21]. Compared with a control group without dysphonia, MTD patients had a significantly larger magnitude adaptive response to changes in auditory feedback, suggesting some type of dysfunction or dysregulation between pitch perception and voice production [21]. There were signs of deficits in temporal auditory processing, auditory discrimination, and adaptive responses in those with voice disorders, compared with those without [6].

Despite some evidence that voice disorders are associated with auditory processing problems, there are several studies which show discrepant results. Davis and Boone [22] compared PD and tonal memory between 30 adult patients with hyperfunctional voice disorders and 30 control participants and showed no significant differences between the two groups. However, there were participants who demonstrated difficulties in PD or remembering a tonal sequence [22]. Another study showed no relationship between PD and voice production in children with and without vocal nodules [23]. The above-mentioned literature has therefore shown conflicting findings related to the association between PD and voice disorders.

In patients with neurological voice disorders, some studies have also reported dysfunctional auditory perception. In spasmodic dysphonia, a neurological voice dystonia of cortical origin, dysfunctional sensory-motor processing was shown when these patients were presented with altered pitch feedback [24]. Patients with unilateral vocal fold paralysis had reduced auditory-processing ability and vocal motor function compared with healthy controls after surgical vocal fold augmentation procedures, as well as differences in the neural areas associated with vocal motor function [25,26]. These studies provide evidence for the impact of damage to the lower motor neuron pathways, involved in production impacting the upper motor neuron pathways (i.e., cortical) involved in perception. Given that vocal production ability shares some neurological pathways in tasks such as speech and musical processing [27,28], it is reasonable to hypothesize that dysfunction in voice production might have effects on PD. Clarifying whether there is such a link between voice quality and pitch perception would be the basis to deliver relevant/specific perception training in parallel to voice restoration/treatment.

We used the Newcastle Assessment of Pitch Discrimination (NeAP) [29] as part of a routine comprehensive assessment of voice function and auditory discrimination. In a previous study, this tool was shown to be reliable and clinically applicable [30]. The aims of the present study were to (1) examine PD characteristics in patients with voice disorders in comparison with non-voice-disordered speakers; and (2) evaluate the value of PD testing in differentiating voice-disordered patients from non-disordered speakers. The overall purpose was to provide clinical data on the use of PD testing in voice-disordered patients to determine the need to pay attention to patient’s auditory perception function for successful voice treatment and provide insight into voice control mechanisms and pathogenesis of voice disorders.

## 2. Materials and Methods

### 2.1. Study Design

This was a cross-sectional study where both voice and auditory discrimination data were collected at a teaching voice clinic at The University of XX. The clinic performed comprehensive standardized voice assessment, including PD testing.

### 2.2. Participants

#### 2.2.1. Voice-Disordered Groups

There were 71 patients (54 females and 17 males) with a confirmed diagnosis of primary or secondary MTVD or a neurological voice disorder. The mean age was 38.5 years (standard deviation, SD = 15.5 years, range = 18–82). Five (7.0%) were vocal performers, 29 (40.8%) were professional voice users, and 37 (52.1%) worked in other occupations. All patients were diagnosed by a laryngologist following conduction of standardized multi-dimensional voice assessment protocols in the University of XX’s voice clinic. Diagnosis was based on patient-reported outcome measures, such as the Voice Handicap Index (VHI-10) [31], speech language pathologist’s (SLP) voice assessment, voice recordings for acoustic analysis, and videostrobolaryngoscopy. There were no patients with hearing impairments as confirmed through audiometric screening (i.e., passing 20-decibel threshold in a pure-tone at 500 Hz, 1 kHz, 2 kHz, and 4 kHz). Participants were excluded if there were self-reported symptoms or clinical signs of speech disorders, cognitive impairments, neurodegenerative conditions, or hearing loss.

In the voice-disordered group there were two sub-groups: MTVD and neurological voice disorder. Table 1 shows patient numbers in each group. In the MTVD group, 26 were diagnosed as primary MTVD and 24 had secondary MTVD with lesions deemed related to phonotrauma such as vocal nodules, pre-nodular swellings, and mucosal thickening. There were 21 patients with neurological voice disorders including vocal fold paresis (*n* = 11), vocal fold paralysis (*n* = 4), tremor (*n* = 3), and laryngeal dystonia (*n* = 3). No patient in the neurological disorder group had Parkinson’s disease, other neurodegenerative, or neuro-cognitive problems. There was a total of 45 voice-disordered patients with a musical training background and 26 without a musical training background.

#### 2.2.2. Control Group

There were 80 participants, all female, with a mean age of 23.5 years (SD = 4.3 years, range = 18–40). All were speech language pathology students. They self-reported as having no voice problems at the time of the study and underwent voice screening using a case history questionnaire and the VHI-10 [31]. Inclusion criteria included no current voice symptoms, VHI-10 < 7.5 [32], normal hearing, and no current upper respiratory problems. Two certified practicing speech language pathologists perceptually assessed their voices using a standardized protocol and confirmed that their voices were non-dysphonic.

Participants in both groups completed a case history questionnaire to determine history of voice disorders, current voice problems, language backgrounds, musical background, and voice/musical training. Musical background was defined as having formally practiced a musical instrument for at least a year past the 5 years of age.

### 2.3. Voice Assessment

Mean VHI-10 score for the voice-disordered group was 20.48 (SD = 10.34, 95% confidence interval, CI = 18.03–22.93), which was above the cut-off score for a voice disorder (>7.5) [32]. Mean VHI-10 score for the control group was 2.28 (SD = 2.03, 95% CI = 1.82–2.73) which fell within the non-disordered range [32].

Acoustic analyses were performed as part of the voice assessment protocols on standardized vocal tasks (middle three seconds of sustained vowel /a/, the third CAPEV phrase [33], and the 2nd and 3rd sentences of the Rainbow Passage [34]). Acoustic measures analyzed for each participant included the harmonics-to-noise ratio (HNR), and cepstral/spectral index of dysphonia (CSID) [35,36]. Acoustic voice data for the groups are presented in Table 2.

### 2.4. Pitch Discrimination Testing

#### 2.4.1. Pitch Discrimination Testing Tool

We used the NeAP [29], which is a two-tone computer-based PD task, where listeners are required to stipulate which tone of a given pair is higher in pitch, or whether they are the same. One study reported on the use of this tool in assessing PD [30], showing it to be reliable, with a moderate to good prediction value in ascertaining one’s musical background.

#### 2.4.2. Protocols

All PD tasks were performed in a sound-protected room with ambient noise measured between 50 and 55 dB sound pressure level (SPL) to avoid effects of noise on auditory discrimination. The NeAP program included 20 tone pairs of sine waves. Each tone pair had a lower frequency tone and a higher frequency tone, with a range of pitch differences between the lower tone and higher tone (Appendix A). The lowest and highest frequency of the lower tones was 123.47 Hz and 293.66 Hz, respectively. The lowest and highest frequency of the higher tones was 130.81 Hz and 311.13 Hz, respectively. The pitch differences between tone pairs ranged from 2.29 Hz to 32.03 Hz (29.98 to 200.01 cents). One semitone is equal to 100 cents.

The tone pairs were played on a Dell computer (Latitude 7280) via two speakers (Harman/Kardon HK645) calibrated to 65.0–65.2 dBA hearing level (HL). Hearing level was measured at 5 cm lateral to the external ear meatus using a lingWAVES sound pressure level meter II model IEC 651. The participant was seated 1 m away equidistantly from the speakers. Participants completed the default protocol of the NeAP program. No training or trial was provided apart from instructions to listen to the tone pairs and to indicate which tone was higher in pitch or if the pitch sounded the same. Participants provided their responses by clicking on one of three buttons on the computer screen. Each button represented ‘tone 1 was higher’, ‘tone 2 was higher’ or ‘both tones were the same’. The 20 tone pairs were presented a second time in a new random order in the same session for reliability analysis. The duration of each tone was 300 milliseconds (ms) and the pause between any two tones was 500 ms. The procedure lasted on average 6 min. The percentage of accurate responses was calculated for each tone pair by dividing the number of accurate responses by the total responses for that tone pair. Outcome measures included the percentage of accurate responses (%) and the mean PD threshold (cent) of correct responses.

### 2.5. Statistical Analysis

Statistical analyses were completed using SPSS 28.0 [38] and MedCalc 20.014 [39]. Data were checked for normal distribution. Intraclass correlation coefficients (ICC) [40] were used to determine the level of agreement between the first and second (repeated) PD responses. ICC was calculated using a two-way mixed model consistency type and single measure analysis [ICC (3,1)]. To help interpret reliability, ICC < 0.5 indicates poor correlation, 0.5–0.75 moderate, 0.75–0.9 good, and >0.9 indicates excellent correlation [41]. Box-Cox transformation was implemented in SPSS for variables with non-normal distribution to obtain a near-normal distribution for parametric tests. A two-way analysis of variance (ANOVA) was used to compare PD scores between groups with a musical background as a fixed factor. Effect sizes are reported as partial Eta squared (η_p_^2^). Effect size of 0.01, 0.1, and 0.25 indicated small, medium, and large statistical effects, respectively [42].

A Receiver Operating Characteristic (ROC) Curve Analysis was calculated to evaluate the value of PD testing in differentiating the voice-disordered groups from the control group. Where there were multiple tests, we used Sidak’s adjustment to the observed *p* values to minimize Type I error. In all calculations, statistical significance testing was two-tailed, *p* < 0.05.

## 3. Results

### 3.1. Reliability of PD Testing

Table 3 shows reliability results for PD testing for all groups. There was good to excellent agreement in PD responses between the first and second trials within all groups.

### 3.2. Percentage of Accurate Responses

#### 3.2.1. Voice-Disordered vs. Non-Voice-Disordered Groups

**Figure 1:**
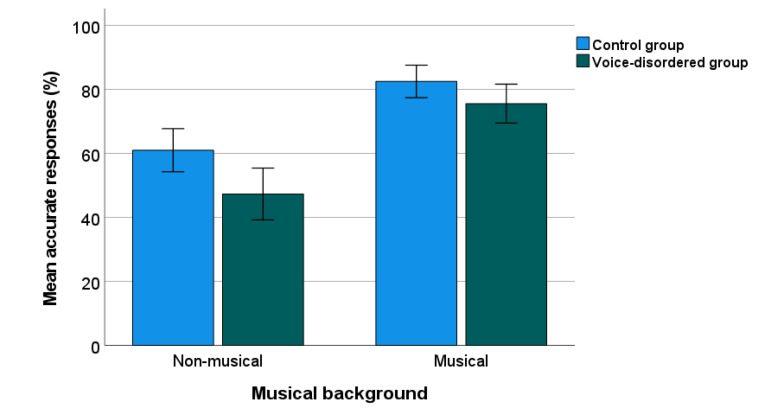
Percentage of PD accuracy in voice-disordered and control groups. Error bars indicate standard errors.

The percentage of correct responses for the PD test is shown in Figure 1. A two-way ANOVA was calculated to compare the correct scores between the voice-disordered group (*n* = 71) and the control group (*n* = 80). Musical background was included as a factor given previous findings of better PD in people with a musical background than those without [12,13]. There were significant effects of group, (F(1, 147) = 9.97, *p* = 0.002, η_p_^2^ = 0.064), and musical background, (F(1, 147) = 57.94, *p* < 0.001, η_p_^2^ = 0.28), but there was no significant interaction (*p* = 0.31). The mean (95% CI) of the percentage of accurate responses was lower by 10.32% (3.86–16.78) in the voice-disordered group compared with the control group (*p* = 0.002).

#### 3.2.2. Sub-Group Comparisons

**Figure 2:**
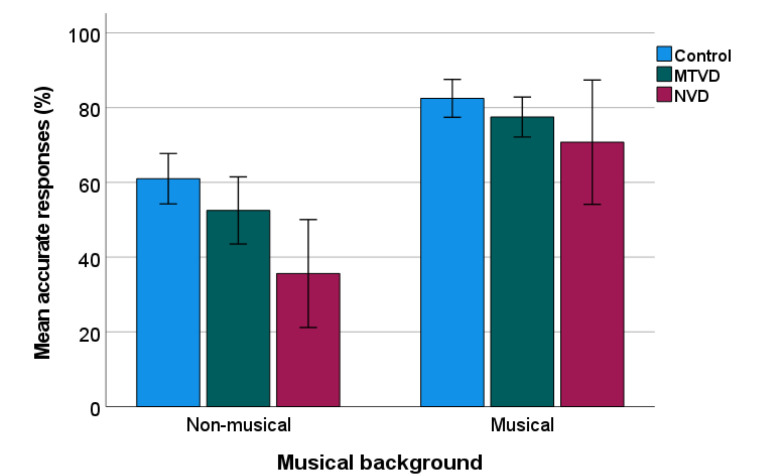
Percentage of PD accuracy in sub-groups. Error bars indicate standard errors. MTVD: muscle tension voice disorder; NVD: neurological voice disorder.

Figure 2 shows the percentage of correct PD responses for sub-groups. Sub-group comparisons were calculated using a two-way ANOVA, comparing across three groups (control, MTVD, neurological) and the two backgrounds (musical, non-musical). Again there was a significant effect of group (F(2, 145) = 7.632, *p* < 0.001, η_p_^2^ = 0.095) and musical background (F(1, 145) = 52.130, *p* < 0.001, η_p_^2^ = 0.264) but no significant interaction (*p* = 0.376).

Post-hoc test using Sidak’s adjustment to the *p* values showed that compared with the control group, the mean of percentage of accurate response was significantly lower by 18.55% (95% CI = 6.77–30.33%) in the neurological group (*p* < 0.001), but not in the MTVD group (mean difference = 6.75%, 95% CI = −1.93–15.43%, *p* = 0.176). The two voice-disordered groups were not significantly different (Mean difference = 11.8%, 95% CI = −0.81–24.41%, *p* = 0.074).

There was no statistical difference (t = 0.153, *p* = 0.879) in the percentage of correct responses (%) between the primary MTVD (n = 26; mean = 68.08, SD = 23.24) and secondary MTVD groups (*n* = 24, mean = 68.96, SD = 17.38).

### 3.3. Pitch Discrimination Threshold

#### 3.3.1. Voice-Disordered vs. Non-Voice-Disordered Groups

**Figure 3:**
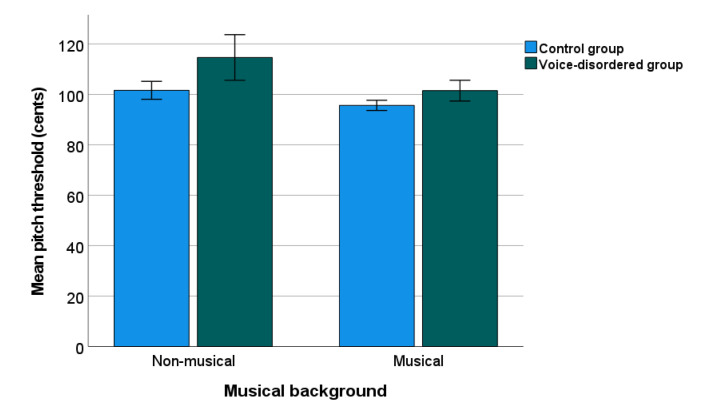
Pitch discrimination threshold in voice-disordered group and control group. Error bars indicate standard errors.

Figure 3 shows the PD threshold data for the voice-disordered (Mean = 108.08 cents) and control groups (Mean = 98.65 cents) by musical background. For statistical analysis, the mean PD threshold for each participant were Box-Cox transformed due to non-normal distribution. A two-way ANOVAs as reported for PD, showed significant effects of group (F(1, 147) = 16.704, *p* < 0.001, η_p_^2^ = 0.102) and musical background (F(1, 147) = 17.212, *p* < 0.001, η_p_^2^ = 0.105), but no interaction (*p* = 0.122). Overall, the PD threshold in voice-disordered patients was 9.43 cents higher than that in the control group.

#### 3.3.2. Sub-Group Comparisons

**Figure 4:**
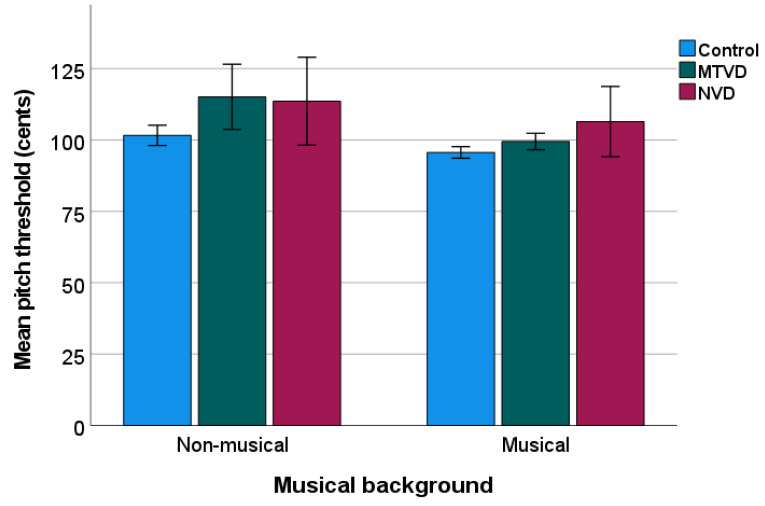
Mean pitch discrimination threshold of sub-groups. The lower pitch threshold, the better discrimination ability. Error bars indicate standard errors. MTVD: muscle tension voice disorder; NVD: neurological voice disorder.

Figure 4 shows the data of the mean PD threshold by sub-groups. Descriptively, mean PD threshold (cents) was higher in each voice-disordered group (MTVD = 105.12; neurological voice disorder = 109.18) than in the control group (Mean = 98.65).

A two-way ANOVA showed significant sub-group (F(2, 145) = 8.723, *p* < 0.001, η_p_^2^ = 0.107) and musical background (F(1, 145) = 12.735, *p* < 0.001, η_p_^2^ = 0.081) effects, but there was no interaction (*p* = 0.163). Post hoc comparisons showed that the PD threshold was significantly higher in both the MTVD group (by 8.66 cents, *p* = 0.003) and neurological voice disorder group (by 11.38 cents, *p* = 0.004), than in the control group. The two voice-disordered groups did not differ (*p* = 0.848).

Pair-wise comparison across sub-groups of the same musical background showed that in the non-musical background group, the mean PD threshold was significantly higher for the MTVD group than for the control group (by 13.49 cents, *p* = 0.002), but there were no differences between the neurological voice disorder group and control group (*p* = 0.080). In the musical background group, the mean PD threshold in the neurological voice disorder group was 10.806 cents higher than that in the control group (*p* = 0.047) whilst this measure was not statistically different between MTVD and controls (*p* = 0.570).

The mean (SD) of the PD threshold (cents) of the primary MTVD and secondary MTVD groups was 105.66 (22.59) and 104.53 (9.54). An independent samples *t*-test showed no statistically significant difference in the PD threshold between primary and secondary MTVD groups (t = 0.235, *p* = 0.816).

The Pearson’s correlation coefficients calculated using the combined sample size of both voice-disordered and control groups (*n* = 151) showed significant correlations between the percentage of correct responses and mean PD threshold (r = −0.695, *p* < 0.001), median pitch threshold (r = −0.483, *p* < 0.001), and minimal pitch threshold (r = −0.488, *p* < 0.001). These implied that the accuracy of responses was associated with the size of the pitch intervals of tone pairs.

### 3.4. Predictive Value of PD Testing in Differentiating Voice-Disordered from Control Groups

**Figure 5:**
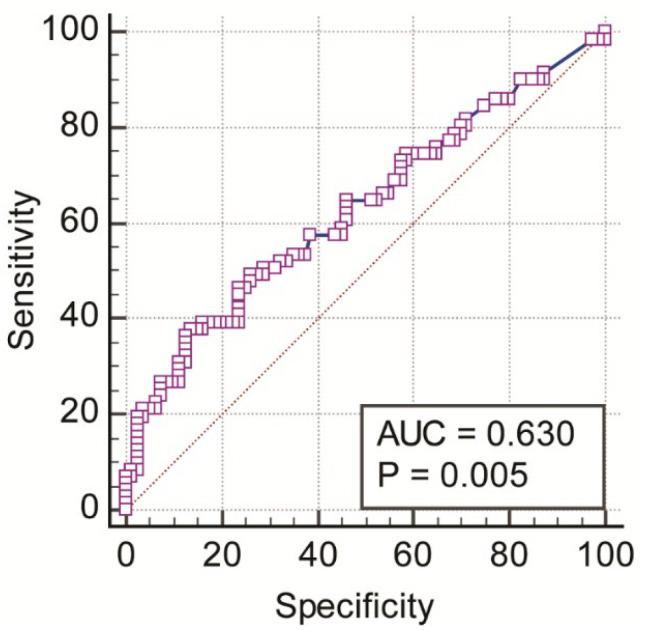
ROC curve for mean pitch discrimination threshold (cents).

An ROC curve (as shown in Figure 5) was analyzed to evaluate the predictive value of PD testing in differentiating the voice-disordered group from the control group. This measure significantly differentiated the two groups (area under the ROC curve, AUC = 0.630, 95% CI = 0.547–0.707, Z-statistic = 2.828, *p* = 0.005). With a Youden index (J) of 0.243 and the associated cut-off value >106.28 cents, this measure differentiated the two groups at a specificity of 86.25% and a sensitivity of 38.03%. At a cut-off of >85.02 cents, sensitivity was 98.59% but specificity was low (2.5%). The cut-off value of >97.45 cents had a balance of both sensitivity (64.79%) and specificity (53.75%).

## 4. Discussion

### 4.1. Pitch Discrimination in Voice-Disordered Patients

As predicted, pitch discrimination accuracy was significantly lower in the voice-disordered group than in the non-voice-disordered group. Based on effect size calculations, the size of this effect was medium. However, with respect to the units of measurement, the difference might be considered small (i.e., 9.43 cents). These differences in pitch discrimination support a previous study [6] showing that patients with behavioral dysphonia had worse pitch perception ability than non-dysphonic speakers. The patients with either MTVD or neurological voice disorder required a larger pitch threshold (above 100 cents) to correctly discriminate the pitch differences compared with the healthy speaker control group (again yielding a medium effect size). Patients with a neurological disorder needed a slightly larger threshold (109.18 cents) than those with a functional voice disorder (105.12 cents), although this difference was not statistically significant.

These results are suggestive of an impairment in auditory discrimination in both functional (MTVD) and neurological voice-disordered individuals. These data are also congruent with work by Abur et al. [4] who showed that the auditory discrimination threshold was significantly larger in patients with hyperfunctional voice disorders (mean = 47 cents, SD = 32 cents) than in control participants (mean = 35 cents, SD = 20 cents). The differences in the auditory discrimination thresholds between our current data and their study likely stemmed from the study design and type of test stimuli. Here, we did not test the just-noticeable-difference (JND) in pitch, but rather used pure tones. In summary, reliable group differences were noted in pitch perception between voice and non-voice-disordered samples, although the clinical relevance of the difference remains to be studied.

In the voice-disordered group, patients with a neurological voice disorder did not show statistically significantly poorer PD than those with MTVD. This suggests voice disorder types and/or dysphonic severity may not be linked to the auditory perception function. This finding appeared to agree with observations by Abur et al. [4] who found no relationship between the overall severity of dysphonia and auditory discrimination threshold. It is important to note that their study [4] only included patients with hyperfunction voice disorders, which might have had smaller range of vocal dysfunction than in our study.

The pitch interval of the pure tones used in the PD testing tool (NeAP) ranged between 29.98 and 200.01 cents. At the smallest pitch interval, the accurate responses for control, MTVD, and neurological group were 29 (19.2%), 18 (11.9%), and 9 (6.0%), respectively. This suggests that the voice-disordered group, particularly the neurological group, had more difficulties discriminating small pitch intervals than controls. We recommend that in future work the JND for different types of voice disorders with different severity should be investigated. This would help to further understand the impact of voice disorders on the minimum pitch difference that a patient can detect, and explore the relationship between voice perception and production.

It is believed that aberrant auditory discrimination plays a role in the pathogenesis of hyperfunctional voice disorder [4]. Current neural models of voice/speech production can be used to explain the poorer PD in those with a voice disorder. In the first place, auditory dysfunction may occur first. The DIVA neural model of phonation [3] states that the control of voice production includes two components: feedforward control (motor components) and feedback control (auditory and somatosensory targets). When the auditory discrimination system is dysfunctional, the ability to detect the mismatch between the expected and real feedback would be decreased. Consequently, this would lead to suboptimal use of the laryngeal motor system in phonation due to the feedforward system failing to update the corrective motor plan provided by the auditory feedback system [4]. This explanation appears to be applicable to MTVD and is supported by previous findings on the mismatch between the auditory-motor control system in patients with hyperfunctional voice disorders [4,5].

In patients with a neurological voice disorder, the model of neural plasticity [43] might explain the poorer PD compared with the non-voice-disordered controls. In patients with neurological voice disorders, neural plasticity may explain the adjustment (increase) in the auditory response threshold to allow for the variability in motor response. Neuroplastic models are well-known when explaining voice and laryngeal syndromes that involve a sensory pathway dysfunction such as the irritable larynx syndrome [44] or the laryngeal hypersensitivity syndromes [45]. A similar neuroplastic process may exist in those with a neurological voice disorder. Increasing the auditory discrimination threshold would benefit the auditory-motor control system in that auditory feedback would be less sensitive to feedback errors and the feedforward system would be less likely to provide motor commands that exceed the capability of the neurologically impaired laryngeal motor system. This model provides an explanation for shifting internal PD thresholds, or other auditory discrimination/perception thresholds to adapt to a worsening voice quality. Over time, if laryngeal coordination is worsened, further feedback would be added to the system, exacerbating the threshold sensitivity. Eventually, there may be more adaptive adjustments in auditory-motor control system, leading to compensatory/suboptimal laryngeal muscle use, or compensatory hyperfunction.

Musical background was factored in the between-subjects analysis due to its known impact on pitch perception. Despite overall differences between individuals in terms of musical background improving pitch discrimination, supporting previous research [12], there were no interactions involving the voice group. Group effects related to musical training background were descriptively similar to those due to voice for pitch accuracy, but for pitch threshold, musical background appeared to have larger and more reliable impacts on pitch perception than voice disorder.

The non-significant interaction effect between groups and musical background in this study was surprising given previous research indicating that both musicians and singers have a greater ability to compensate for pitch disturbances [46,47]. The above-mentioned mechanisms explaining the reasons for poor pitch discrimination in voice-disordered individuals might bypass or over-ride the well-established reflexes or processes formed in those with musical and/or singing training. This non-interaction between voice groups and musical background also implied that training does allow individuals, regardless of pathology, to improve pitch discrimination.

### 4.2. Predictive Value of PD Testing

Results of the ROC curve analyses showed that the PD threshold had a predictive ability to discriminate between voice-disordered and control groups. This suggests that it is possible to use PD testing as a method to differentiate a voice-disordered group from non-disordered speakers. It is necessary to develop/revise the PD testing tool to include a wider range of pitch intervals/differences and test its sensitivity and specificity in different levels of dysphonic severity and different voice disorder types. This development will allow validation of its applicability in clinical settings. In the present study, the sensitivity and specificity of this measure were relatively low if a balance between them is used in determining a cut-off value.

Previous research showed that people without musical training required thresholds between 1 and 3 semitones (100–300 cents) to be able to discriminate pitch intervals [48]. In the present study we found that a cut-off of >97.45 cents had a reasonable balance between sensitivity and specificity of testing. However, the relatively low sensitivity and specificity probably resulted from heterogeneity within the voice-disordered groups (i.e., including both functional and neurological voice disorders). We did not perform the ROC analyses separately for the MTVD and neurological voice disorder groups and for the two musical background due to the small sample size of each subgroup. It may also be the case that the current NeAP protocol was not associated with optimal prediction ability given the number of tone pairs used (20) and the range of PD threshold. Smaller thresholds would probably be more likely to differentiate the groups with better sensitivity and/or specificity.

This study had several limitations that should be addressed in future research to help with internal and external validity. Firstly, this was a cross-sectional observational study and not a prospective cohort study. Consequently, this design did not allow the determination of PD of the dysphonic speaker prior to having a voice disorder. Therefore, we cannot state that PD deteriorated in these patients when they acquired a voice disorder. A second issue related to validity, was that the control group comprised all females at a younger age range than the dysphonic group. As auditory perception may vary as a function of age, better matched comparison groups will be needed to determine the size and reliability of any effects due to voice pathology. Lastly, despite its utility and functionality, there is a lack of literature exploring the sensitivity and specificity of the NeAP testing tool in differentiating those with and without voice disorders according to their PD abilities. Further studies are needed to validate this tool for clinical application.

## 5. Conclusions

Here we showed that patients with a voice disorder had poorer PD than non-voice-disordered controls. Patients with MTVD and neurological voice disorders had a lower percentage of accurate PD responses and required larger pitch discrimination thresholds to correctly identify pitch differences between tone pairs. These findings provided more evidence for a possible dysfunction or dysregulation of both auditory discrimination pathways and laryngeal motor control in these voice-disordered groups. The mechanisms for poorer PD might be different between functional/MTVD voice disorders and neurological voice disorders given the differences in the pathogenesis of each disorder type.

PD testing significantly differentiated voice-disordered patients (MTVD and neurological voice disorders) from non-disordered speakers. This finding is important as PD testing can serve as not only a diagnostic tool but also a follow-up tool during the treatment process. Moreover, the fact that musical background significantly distinguished PD ability irrespective of voice disorder, suggests that problems in perception can be overcome with training. These data highlight the need to evaluate both auditory discrimination function and voice quality across the diagnosis, treatment, and follow-up stages for voice disorders. It would be necessary to clarify whether PD changes reflect treatment outcome.

## Figures and Tables

**Table 1:** Number of participants by groups. MTVD: muscle tension voice disorder.

Groups	Musical Background		Total
	No	Yes	
Control	40	40	80
MTVD no lesions	7	19	26
MTVD with lesions	11	13	24
Neurological dysphonia	8	13	21
Total	66	85	151

**Table 2:** Mean (SD) and 95% confidence intervals for the mean of voice data for voice-disordered and control groups. The *p* value indicates significance level of independent *t*-test comparisons of the voice-disordered group (*n* = 71) and control group (*n* = 80) for each measure. HNR: harmonics-to-noise ration; CSID: cepstral/spectral index of dysphonia.

Measures (Normative Values)	Voice-Disordered Group			Control (*n* = 80)	*p*
	MTVD (*n* = 50)	Neurological Voice Disorders (*n* = 21)	All (*n* = 71)		
HNR (dB) (20 dB) [37]	23.90 (4.16) 22.71–25.10	21.36 (7.80) 17.81–24.91	23.14 (5.57) 21.81–24.47	24.90 (2.46) 24.35–25.45	0.016
CSID of vowel (NA)	9.03 (18.17) 3.81–14.25	25.43 (31.79) 10.96–39.90	13.95 (24.08) 8.21–19.69	−10.81 (7.38) (−12.45)–(−9.17)	<0.001
CSID of CAPEV-3 (NA)	−10.18 (20.78) (−16.15)–(−4.21)	1.42 (30.05) (−12.26)–15.10	−6.70 (24.31) (−12.49)–(−0.90)	−16.36 (9.48) (−18.47)–(−14.25)	<0.001
CSID Rainbow Passage (24.27) [36]	17.49 (14.05) 13.45–21.52	21.13 (17.84) 13.01–29.25	18.58 (15.24) 14.95–22.21	−3.16 (19.19) (−7.43)–1.12	<0.001

**Table 3:** Reliability of PD testing. ICC: intraclass correlation coefficient; CI: confidence interval, MTVD: muscle tension voice disorders.

Groups	Single Measures (ICC, 95% CI)	Average Measures (ICC, 95% CI)	*p*
Whole cohort	0.899 (0.863–0.926)	0.947 (0.927–0.961)	<0.001
Control (*n* = 80)	0.880 (0.819–0.921)	0.936 (0.901–0.959)	<0.001
MTVD (*n* = 50)	0.847 (0.746–0.910)	0.917 (0.854–0.953)	<0.001
Neuro (*n* = 21)	0.972 (0.933–0.989)	0.986 (0.965–0.994)	<0.0001

## Data Availability

Data supporting reported results is retained by The University of Sydney in a de-identified form and is confidential under the conditions of the Human Research Ethics Committee of The University of Sydney approval.

## Appendix A

**Table A1:** Frequency of tone pairs of the NeAP.

Tone Pairs	Frequency 1 (Hz)	Frequency 2 (Hz)	Hz Difference	Cent Difference
C3 (130.81) D3(146.83)	130.81	146.83	16.02	200.01
A3 (220.00) B3 (246.94)	220	246.94	26.94	199.99
C4 (261.63) D4 (293.66)	261.63	293.66	32.03	199.94
B2 (123.47) C3 (130.81)	123.47	130.81	7.34	99.97
E3 (164.81) F3 (174.61)	164.81	174.61	9.8	100.00
F3 (174.61) F#3 (185.00)	174.61	185	10.39	100.07
C3 (130.81) C#3 (138.59)	130.81	138.59	7.78	100.02
G3 (196.00) G#3 (207.65)	196	207.65	11.65	99.96
D4 (293.66) D#4 (311.13)	293.66	311.13	17.47	100.04
A3 (220.00) A#3 (233.08)	220	233.08	13.08	99.99
D4 (293.66) D4.5 (302.26)	293.66	302.26	8.6	49.97
F3 (174.61) F3.5 (179.73)	174.61	179.73	5.12	50.03
C3 (130.31) C3.5 (134.64)	130.31	134.64	4.33	56.59
A3 (220.00) A3.5 (226.45)	220	226.46	6.46	50.10
E3 (164.81) E3.5 (169.64)	164.81	169.64	4.83	50.01
D3 (146.83) D3.5 (151.13)	146.83	151.13	4.3	49.97
B3 (246.94) B3.5 (254.18)	246.94	254.18	7.24	50.03
G3 (196.00) G3.5 (201.74)	196	201.74	5.74	49.97
C3 (130.81) C3.3 (133.1)	130.81	133.1	2.29	30.05
F3 (174.61) F3.3 (177.66)	174.61	177.66	3.05	29.98
